# 

**Published:** 1856-06

**Authors:** 


### INDEX TO VOLUME TWELFTH.

For original communications, look for the names of the authors as well as the titles of the subjects.

For selected and editorial articles, look for the titles of the subjects only.

For notices of books, look for names of authors.

### ILLUSTRATIONS.

Buffum's Obstetrical Extractor......................... Pages 229, 230
Hamilton's Case of Traumatic Aneurism..................... Page 470
Bauer's Wire Cloth Splints....................... Pages 665, 666, 667

### A.

PAGE.
Abscess, Pulmonary. By A. M. Leonard, M. D., Clarkson, Monroe Co., N. Y.,...................... 14
Academy of Medicine, New York,. 508
Annual Address of the President of the Erie County Medical Society. By Wm. Van Pelt, M. D.,......... 554
Address of President of Illinois State Medical Society,................ 354
Address, review of above,.......... 372
Agassiz, Address of, at Albany,...... 249
Agnew's Practical Anatomy,........ 351
American Medical Authors,......... 246
Anniversary of Oneida County Medical Society,..................... 183
Anatomy, Agnew's Practical,....... 351
Anatomy, Wilson's Dissector,....... 351
Analysis of Homœopathic Remedies, 365
Aneurism, Hamilton on Femoral Traumatic,...................... 466
Asylum for Inebriates,............... 383
Asthma,........................... 447
Association, Proceedings of Buffalo Medical,....20, 65, 141, 203, 290, 340 412, 484, 541, 625, 676, 716
Association, American Medical,.... 38 430, 623
Association, Presidency of American Medical,......................240, 379
Ascites, Chronic. By E. W. Cheney, M. D., Canandaigua, N. Y.,...... 18
Atresia Vaginæ,.................. 495

### B.

Barlow's Practice of Medicine,..... 88
Baths, Chemical,.................. 29J
Baker, M. D., Charles H., on Dislocation of Hip,..................... 624
Bauer, M. D., Louis, on Use of Wire Cloth in Surgery,............... 663
Beck's Materia Medica,........... 572
Bedford on Diseases of Women and Children,....................... 682
Boarding School Nuisance.......... 182
Brown on Surgical Diseases of Women,.......................... 215
Brigham Hall, Canandaigua,....... 503
Buffalo Medical Association Proceedings of,.......20, 65, 141, 203, 290, 340 412, 484, 541, 625, 676, 716

PAGE.

Buffalo Hospital, Report of Surgeons of, ... 61
Buffalo, Mortality of. By C. D. Dayton, M. D., ....30, 94, 153, 218, 297, 352 427, 490, 567, 628, 683
Buffalo Annual Report of Mortality, by C. L. Dayton, ... 689
Buffalo, University of, ... 244, 635
Buffalonian at the West, ... 630
Budd on Stomach, ... 28
Bubonocele, Case of, by H. P. Strong, M. D., Beloit, Wis., ... 611

C.

Cæsarian Section, ... 309
Carpenter on the Microscope, ... 348
Cathartics in Dysentery, by O. C. Gibbs, M. D., Fréwsburg, Chaut. Co., N. Y., ... 481
Cancer, Landolfi's Treatment, ... 494
Calculus, Renal, by C. M. Hewitt, M. D., Geneva, N. Y., ... 623
Cheney, M. D., E. M., of Canandaigua, A Case of Ascites, ... 18
Chemistry, Lehmaun's Physiological 91
Chemistry, Gregory's Inorganic, ... 426
Chloroform in Surgery, ... 103
Cholera Statistics, ... 317
Chlorate of Potassa, ... 500
Clinical Lecture's, Bedford's, ... 682
Correlation of Physical Sciences.. . 34
Compend, Neill and Smith's, ... 93
Colvin, M. D., Darwin, on Fracture of Skull, ... 617
Convulsions, Puerperal, by James C. Reeves, M. D., of Phillippi, Va.,.. 129
Croup and its Treatment, ... 32
Croup, Essay on, ... 162, 220
Croup, Tracheotomy in, ... 486
Curling on Diseases of the Testis... 217
Cyanosis, Case of, by J. T. Jamison, M. D., South Otselic, Chenango Co., N. Y., ... 19

D.

Dayton, M. D., Charles L., Report of Mortality in Buffalo, ....30, 94, 153, 218 297, 353, 427, 490, 567, 628, 683
Dayton, M. D., Charles L., Annual Report of, ... 689

PAGE.

Diary for 1857, ... 448
Deaths in Buffalo, ....30, 94, 153, 218, 297 352, 427, 490, 567, 628, 683
Dislocation of Cervical Vertebræ, by E. R. Maxson, M. D., Geneva, N. Y. 479
Dislocation of Hip Joint, by C. H. Baker, M. D., ... 624
Dropsy, Ovarian, by J. T. Jamison, M. D., South Otselic, N. Y., ... 206
Doctor in the Witness Box ... 115
Draper's Physiology, ... 295
Dunglison's New Remedies, ... 627
Dysentery, Analysis of Eleven Cases of, by Austin W. Nichols, M. D.,. 130
Dysentery, Cathartics in, by O. C. Gibbs, M. D., of Frewsburg, N. Y.. 481

E.

Editors, Convention of Medical, ... 702
Epilepsy, ... 685
Erie County Medical Society, ... 122 446, 574
Erie Co. Medical Society, Address of Dr. Van Pelt, President, ... 554
Erie County Poor House, ... 375
Erysipelas and Rupture of the Stomach, by D. W. Young, M. D., Aurora, Ill., ... 471
Europe, Letter from, ... 698
Excision of Elbow Joint, ... 105
Exopthalmos and Exopthalmia, by S. B. Hunt, M. D., ... 199
Extraction of Needles, ... 492
Extractor Obstetrical, ... 228

F.

Fevers, Blending of Periodical and Continued, by Austin Flint, M. D., Buffalo, N. Y., ... 1
Fevers, Blending of, by Austin W. Nichols, M. D., Buffalo, N. Y., ... 81
Fevers, Blending of, by O. C. Gibbs, M. D., Frewsburg, N. Y., ... 148
Fever, Intermittent, Treatment of, by Austin W. Nichols, M. D., ... 460
Fever, Yellow, by R. LaRoche, M.D., 242
Fever, Typhoid, in Virginia, by Jas. E. Reeves, M. D., Phillippi, Va.,.. 257
Female Medical Education, ... 112
Flint, Prof. Austin, on Fevers, ... 1

PAGE.
Fistula-Vesico Vaginal, by Prof. F. H. Hamilton,.................. 150
Fractures, Cases of, by Prof. F. H. Hamilton, M. D., Buffalo, N. Y.,.. 278
321, 385, 449, 513, 577, 641
Fracture of Skull, by Darius Colvin, M. D., Clyde, N. Y.,.............. 617
Functions of Spinal Marrow,........ 118
Functions of County Medical Societies,.......................... 187

### G.

Gibbs, Dr. O. C., of Frewsburg, Chaut. Co., N. Y., on Blending of Fevers,........................ 148
Gibbs on Dysentery,................ 481
Gardner on Sterility,............... 216
Gluck, Dr. Isodor, Letters from,.264, 698
Gregory's Chemistry,............... 426

### H.

Hamilton, Prof. F. H., of Buffalo, Cases of Fractures,....278, 321, 385
449, 513, 577, 641
Hamilton, Case of Occlusio Vaginæ,. 198
Hamilton, Case of Vesico-Vaginal Fistula,......................... 150
Hamilton, Case of Traumatic Femoral Aneurism,..................... 466
Head, Injuries of, by J. T. Jamison, M. D, South Otselic, Chenango Co., N. Y.,................ 521
Hewitt, M. D., Charles M., of Geneva, N. Y., Renal Calculus.............. 623
Headaches, Wright on,.............. 93
Hospital Report,..................... 61
Hospital Clinical Reports, by A. W. Nichols, M. D.,..............667, 673
Hospitals of Vienna, Holmes on.... 76
Holmes, M. D., Edward L., on Vienna Hospitals,......................... 76
Homœopathic Remedies,........... 365
Holland's Medical Notes,........... 680
Hunt, Prof. Sanford B., on Exopthalmos,.......................... 199
Hunt, Prof. Sanford B., on Sun Stroke,.......................... 23

### I.

PAGE.
Illinois State Med. Society, Address of President of,..............354, 372
Inorganic Chemistry, Gregory's,.... 426
Intermittent Fever, Its Modes of Treatment, by A. W. Nichols, M. D., Buffalo, N. Y................ 460
Infanticide, Case of,................ 512
Inflammation and its Local Treatment, by H. P. Strong, M. D., Beloit, Rock Co., Wis.,................. 519
Injuries of the Head, by J. T. Jamison, M. D., South Otselic, Chenango Co., N. Y., .............. 521
Insanity, Moral,.................... 569
Insanity, Medico-Legal,............ 634
Irritation, Gastric,.................. 99

### J.

Jamison, M. D., J. T., South Otselic, Chenango Co., N. Y., Case of Cyanosis,...................... 19
Jamison, M. D., J. T., Case of Ovarian Dropsy,..................... 206
Jamison, M. D., J. T., Case of Injuries of Head,........................ 521

### L.

Leonard, M. D., A. M., of Clarkson, Monroe Co., N. Y., on Pulmonary Abscess,........................ 14
Laceration of the Womb, by E. R. Maxson, M. D., Geneva, N. Y.,.... 154
Landolfi on Cancer,................ 494
Laycock on Medical Observation,... 681
Lehmann's Chemical Physiology,.. 91
Library of R. C. S. England,........ 246

### M.

Maxson, M. D., E. R., of Geneva, N. Y., on Laceration of the Womb,.. 154
Maxson, M. D., E. R., on Dislocation of Vertebræ,...................... 479
Mack, M. D., Theophilus, of St. Catherines, C. W., on St. Catherines Well,....................... 193
Malpractice in Vermont,............ 63
Materia Medica, Beck's,............ 571
Meachem, M. D., Wm. G., on Retention of Urine,.................... 619

PAGE.

Medical Profession,................ 173
Medical Profession in Ancient Times 192
Medical Schools,................. 180
Medical Notes, Holland's,........... 680
Medical News,..................... 120
Medical Societies, Powers of,........ 187
Meetings of Erie County Medical Society,................122, 446, 574
Memoirs, Obstetric, of Simpson,.... 209
Meningitis, Cerebro-Spinal,......... 697
Mercurials, Anti-Syphilitics,........ 703
Miller's Principles of Surgery....... 90
Microscope, Carpenter on,.......... 348
Microscopic Inspection of Retina,... 692
Morphia in Chest Diseases,.......... 96
Moral Insanity,.................... 569
Medico-Legal Insanity,............. 634

N.

Neill and Smith's Compend,....... 93
Neligan on Diseases of Skin,........ 217
Needles, Extraction of,............. 492
Neuralgia, ....................... 502
New Remedies, Dunglison's,........ 627
New Orleans Sanitary Commission. 636
New York Academy of Medicine... 508
Nichols, M. D., Austin W., of Buffalo, N. Y., on Blending of Typhoid and Remittent Fevers,............ 81
Nichols, M. D., Austin W., on Dysentery, ..................... 133
Nichols, M. D., Austin W., on Intermittent Fever,................. 469
Nichols, M. D., Austin W., Clinical Reports,....................667, 673
Notes and Reflections, Holland's,.. 680

O.

Occlusio Vaginæ, by Prof. F. H. Hamilton, M. D.,.................... 198
Obstetric Memoirs,................. 209
Obstetrics, ....................... 573
Obstetrical Extractor,............. 228
Observations, Medical,............ 681
Oneida County Society, 50th Anniversary, ...................... 183
Ovarian Dropsy, by J. T. Jamison, M. D., of South Otselic, N. Y.,... 206
Ovarian Tumour, Removed by Operation, by Nelson Winton, M. D., Havana, N. Y.,................. 415

P.

Pathological Society of New York,. 155
Paraphimosis, ..................... 161
Parrish's Pharmacy,............... 92
Pamphlet Notices,................. 391
Peninsular Journal,.............570, 695
Physical Sciences,................ 34
Physician's Diary,..............254, 448
Physiology, Draper's,............. 295
Poor House Report,............... 375
Profession, Medical,.............. 173
Presidency of American Medical Association,..................240, 379
Pregnancy, Extra-Uterine,.......... 307
Practice, Barlow's,................ 88
Pulmonary Abscess, Case of, by A. M. Leonard, M. D., Clarkson, Monroe Co., N. Y.,.................. 14
Puerperal Convulsions, Case of, by James E. Reeves, M. D., Phillippi, Va., ........................... 129

R.

Reeves, M. D., James E., Phillippi, Va., Case of Puerperal Convulsions,........................... 129
Reeves, M. D., James E., on Typhoid Fever in Virginia,.............. 257
Reviews of Medical Books,......... 316
Report of Erie Co. Poor House,.... 375
Report of C. L Dayton, Health Physician,........................ 689
Reports of Cases in the Medical Ward of the Buffalo Hospital of the Sisters of Charity, during the Session of the Medical College for the Winter of 1856-7, by Austin Flint, M. D., ........................ 718
Retention of Urine, by William G. Meachem, M. D., Warsaw, N. Y.,. 619
Renal Calculus, Case of, by C. M. Hewitt, M. D., Geneva, N. Y.,.... 623
Retina, Inspection of,............. 692
Ricord and his Patient,............ 102

PAGE.

Rupture of the Stomach, Case of, by D. W. Young, M. D., Aurora, Ill.,. 689

S.

Sanitary Commission of N. Orleans,. 636
Shortcomings, .......... 571
Simpson's Obstetric Memoirs,....... 209
Skin, Neligan on the, .......... 217
Skull, Fracture of, Case by D. Colvin, M. D., Clyde, N. Y., .......... 617
Smith's Surgery, .......... 489
Society, Erie County, .......446, 554, 574
Stomach, Budd on, .......... 28
St. Catherines' Well, by Theo. Mack, M. D., St. Catherines, C. W., ....... 193
Sterility, Gardner on, .......... 216
Stomach, Rupture of, Case by D. W. Young, M. D., Aurora, Ill., ........ 471
Strong, Dr. H. P., Beloit, Rock Co., Wis., on Inflammation, .......... 519
Strong, Dr. H. P, on Bubonocele,... 611
Sun Stroke, by Prof. S. B. Hunt, Buffalo, N. Y., .......... 23
Surgery, Miller's, .......... 99
Surgical Diseases of Women, ........ 215
Syncope from Gastric Irritation,.... 99

T.

Testis, Curling on the, .......... 217
Title Mania, .......... 246
Therapeutics, Wood's, .......... 424

PAGE.

Transactions of N. Y. State Medical Society, .......... 86
Transactions of American Medical Association, .......... 430
Trousseau on Tracheotomy in Croup, 486
Typhoid Fever in Northwestern Virginia, by James E. Reeves, M. D., Phillippi, Va., .......... 257

U.

Uterus, Excision of, .......... 301
University of Buffalo, .......37, 244, 635

V.

Van Pelt, M. D., William, Address as President of Erie Co. Society,.... 554
Valerianate of Ammonia, .......... 502
Vagina, Occlusion of, by Prof. F. H. Hamilton, .......... 198
Vagina, Occlusion, Case of, .......... 495
Vesico-Vaginal Fistula, Case of, by Prof. F. H. Hamiton.......... 150
Vertebræ, Dislocation of, by E. R. Maxson, M. D., Geneva, N. Y., .... 479

Y.

Yellow Fever, LaRoche on, .......... 242
Young, Dr. D. W., Aurora, Ill., Case of Erysipelas, and Rupture of the Stomach, .......... 471

*Resignation of Prof. Ackley.*—Professor H. A. Ackley has resigned the chair of Surgery in the Cleveland Medical College. He has held this position from the organization of the school in 1843, and during all that time has been one of its most important supports. Gifted with all the qualities of a great surgeon, he is bold and unflinching in his operations, and has been untiring in his efforts to sustain a splendid surgical clinic.

It is not too much to say that his clinical days were by far the most brilliant feature of that institution. His resignation will, we fear, be a heavy blow to the school. It has been for some time anticipated, but he has heretofore yielded to the solicitations of his colleagues, and continued his labors for the prosperity of the college. It will be very difficult to fill his place with a man equal to him in surgical reputation and industry. In fact it may be doubted whether another man, however able, could call around him such abundant clinical material.

---

*American Medical Association.*—Our readers will find in our pages an ample report of the proceedings of this body at its recent meeting in Detroit. We were unfortunately unable to be present in person, but we learn from other sources that the meeting was a brilliant one, and that the profession and citizens of Detroit entertained their visitors in a most elegant style of hospitality. Several brilliant parties were given on every evening, and all the delegates expressed themselves delighted with their visit to the "City of the Straits."

